# Plant growth strategies are remodeled by spaceflight

**DOI:** 10.1186/1471-2229-12-232

**Published:** 2012-12-07

**Authors:** Anna-Lisa Paul, Claire E Amalfitano, Robert J Ferl

**Affiliations:** 1 Department of Horticultural Sciences, University of Florida, Gainesville, FL, 32611, USA; 2 Program in Plant Molecular and Cellular Biology, University of Florida, Gainesville, FL, 32611, USA; 3 Interdisciplinary Center for Biotechnology Research, University of Florida, Gainesville, FL, 32610, USA

## Abstract

**Background:**

Arabidopsis plants were grown on the International Space Station within specialized hardware that combined a plant growth habitat with a camera system that can capture images at regular intervals of growth. The Imaging hardware delivers telemetric data from the ISS, specifically images received in real-time from experiments on orbit, providing science without sample return. Comparable Ground Controls were grown in a sister unit that is maintained in the Orbital Environment Simulator at Kennedy Space Center. One of many types of biological data that can be analyzed in this fashion is root morphology. Arabidopsis seeds were geminated on orbit on nutrient gel Petri plates in a configuration that encouraged growth along the surface of the gel. Photos were taken every six hours for the 15 days of the experiment.

**Results:**

In the absence of gravity, but the presence of directional light, spaceflight roots remained strongly negatively phototropic and grew in the opposite direction of the shoot growth; however, cultivars WS and Col-0 displayed two distinct, marked differences in their growth patterns. First, cultivar WS skewed strongly to the right on orbit, while cultivar Col-0 grew with little deviation away from the light source. Second, the Spaceflight environment also impacted the rate of growth in Arabidopsis. The size of the Flight plants (as measured by primary root and hypocotyl length) was uniformly smaller than comparably aged Ground Control plants in both cultivars.

**Conclusions:**

Skewing and waving, thought to be gravity dependent phenomena, occur in spaceflight plants. In the presence of an orienting light source, phenotypic trends in skewing are gravity independent, and the general patterns of directional root growth typified by a given genotype in unit gravity are recapitulated on orbit, although overall growth patterns on orbit are less uniform. Skewing appears independent of axial orientation on the ISS – suggesting that other tropisms (such as for oxygen and temperature) do not influence skewing. An aspect of the spaceflight environment also retards the rate of early Arabidopsis growth.

## Background

It is well known that plant growth patterns are influenced by a variety of stimuli and the responses to stimuli such as gravity have been explored and documented for over a century. In the past few decades it has been shown that the circumnutating “behavior” in plants, first described by Charles and Francis Darwin [1] and most recently revisited in [2] is primarily due to radially asymmetric growth in the elongating organs, which is in turn influenced by a variety of environmental stimuli (e.g. [3-7]). Of particular interest to us are the fundamental responses of plants to the unique environment of spaceflight, specifically the skewing and waving response of roots of *Arabidopsis thaliana* (Arabidopsis) in microgravity on board the International Space Station.

Plant roots on Earth exhibit waving, the regular, periodic, nutational change in the direction of the root tips during growth. Waving is thought to be associated with perception and avoidance of obstacles and is thought to be dependent on correct gravity sensing and responsiveness, as mutations in genes associated with gravity sensing disrupt the signal transduction mechanism and abolish the waving phenotype [8,9]. The movement of waving is a growth phenomenon, the result of altered cell file rotations (CFR) about the main axis of root growth. Waving is recorded over time as sinusoidal growth patterns as roots grow along an agar-based surface, with a near-circadian periodicity.

Plant roots may also exhibit skewing, the term given to the longer duration, slanted progression of roots growing along a near-vertical surface, and which may be related to the same CFR that directs waving. In certain cultivars of Arabidopsis an endogenous structural asymmetry results in a rotation of the root tip, which then manifests as right-handed slanting (or skewing to the left in most images) [10]. Microscopic inspection of the tip of skewing roots reveals evidence of CFR around the root axis. Roots that do not skew, either due to genotype or environment (as in roots embedded in agar) exhibit little CFR, while in genotypes prone to skewing there is a positive correlation with degree of incline to the extent of CFR. Skewing varies in its intensity among ecotypes. Ecotype Columbia (Col-0) shows almost no skewing, while Landsberg *erecta* (L*er*) and Wassilewskija (WS) ecotypes exhibit growth that slants between 17 (L*er*) and 19 degrees (WS) from the vertical [10-12].

It has long been known that a large part of skewing is a surface-dependent phenomenon. Roots growing embedded in agar do not skew [10]. In addition skewing seems to involve a complex interdependency of gravity, light and other environmental gradients; Arabidopsis (WS) plants exhibit right-handed skewing regardless of the direction of light, source of nutrients or water, and even when the gravity vector is disrupted artificially, as with clinorotation. Indeed, it appears that the primary point of influence of external factors (gravity, light, water, nutrients) is through the means by which that factor, or combination of factors, contributes to the quality of interaction between root and growing surface. [8,10]. This phenomenon is easily visualized with the factor of gravity in Arabidopsis WS plants. Where the surface interaction is uniform, such as when a root is growing completely within an agar matrix, the root grows virtually straight along the gravity vector. Growth along a vertical surface results in a slight skew to the right. However, when a root grows along the surface of a 45° inclined plane, the force of gravity increases the force of contact between root and growing surface, which results in enhanced skewing and waving as the root grows along the surface. When a root tip reaches a surface perpendicular to the gravity vector, it grows in a clock-wise spiral along that new surface.

But what happens when you remove gravity as a factor completely? The only environment that can truly remove the influence of gravity in an experimental setting is the orbital microgravity environment, such as provided by Space Shuttle flights or time on the International Space Station (ISS). There have been numerous plant spaceflight experiments that have focused on gravity sensing and accompanying morphometric traits (e.g.: [13-22]) and reviewed in [23-25]. A recurring conclusion is that gravity dominates tropisms and morphology in terrestrially-grown plants and that removing gravity from the equation reveals unique aspects of inherent patterns of cell growth and development. The effect of the microgravity environment varies among plant species and even among cultivars of the same species; in the absence of light, the roots of Arabidopsis cultivar Landsberg demonstrate an inherent skew to the right [16], while cultivar Columbia appears to grow randomly on orbit [22].

We present here detailed time-lapse plant growth and development data from an orbital experiment following the growth and development of two cultivars of Arabidopsis (Wassilewskija; WS and Columbia; Col-0) in specialized spaceflight hardware designed for continuous visual data collection. The data are from Run 3 of the APEX-TAGES spaceflight experiment launched on STS-130 in February, 2010 and delivered to the International Space Station (ISS). The APEX-TAGES experiment focused on the discovery of morphological data over time, as well as molecular and physiological changes in Arabidopsis in response to the orbital environment. APEX-TAGES plants were grown on nutrient-agar plates, allowing a clear examination of root growth, waving and skewing along the surface. The plants were grown in the Advanced Biological Research System (ABRS), which has an imaging system that collected digital photographs of WS and Col-0 from germination to near maturity (12–15 days) while on the ISS.

## Results

### Novel imaging hardware records distinctive patterns of growth for spaceflight and ground control plants

The imaging data from orbit revealed that the Arabidopsis cultivars Wassilewskija (WS) and Columbia (Col-0) were different in their responses to spaceflight. There were fundamental differences in root growth and development on orbit compared to control sets grown in identical hardware on the ground in the Orbital Environment Simulator (OES). However, although distinct differences between Flight and Ground Control plants were evident, both cultivars recapitulated the general patterns of growth well established for WS and Col-0 cultivars in terrestrial studies; WS skewed strongly to the right while Col-0 displayed minor skewing to the left.

The foundational growth medium for Arabidopsis plants on orbit was phytagel in standard 100mm square Petri plates. This configuration was chosen for several reasons. First, the petri plates are a standard physical format that has been used for numerous ground based studies on skewing and waving, as well as for previous spaceflight experiments [26-28]. Phytagel is a solid, non-friable, water and nutrient delivery system ideal for microgravity environments in that water and nutrient delivery are passive and consistent. Moreover, working within this common physical format allows the spaceflight hardware to be tasked for other common biological experiments. Second, Arabidopsis growth on these plates allows for excellent gas exchange, complete hydration and adequate nutrition for the 12–15 days of the studies. In addition, roots grow along the surface of the phytagel, creating a uniform focal field for photographic records and a substrate by which surface-related phenomenon, such as skewing and waving, could be evaluated. Third, properly treated seeds on the plates can remain dormant under spaceflight ambient conditions, germinating only when mounted within the growth hardware and exposed to light to start the experiment.

**Figure 1:**
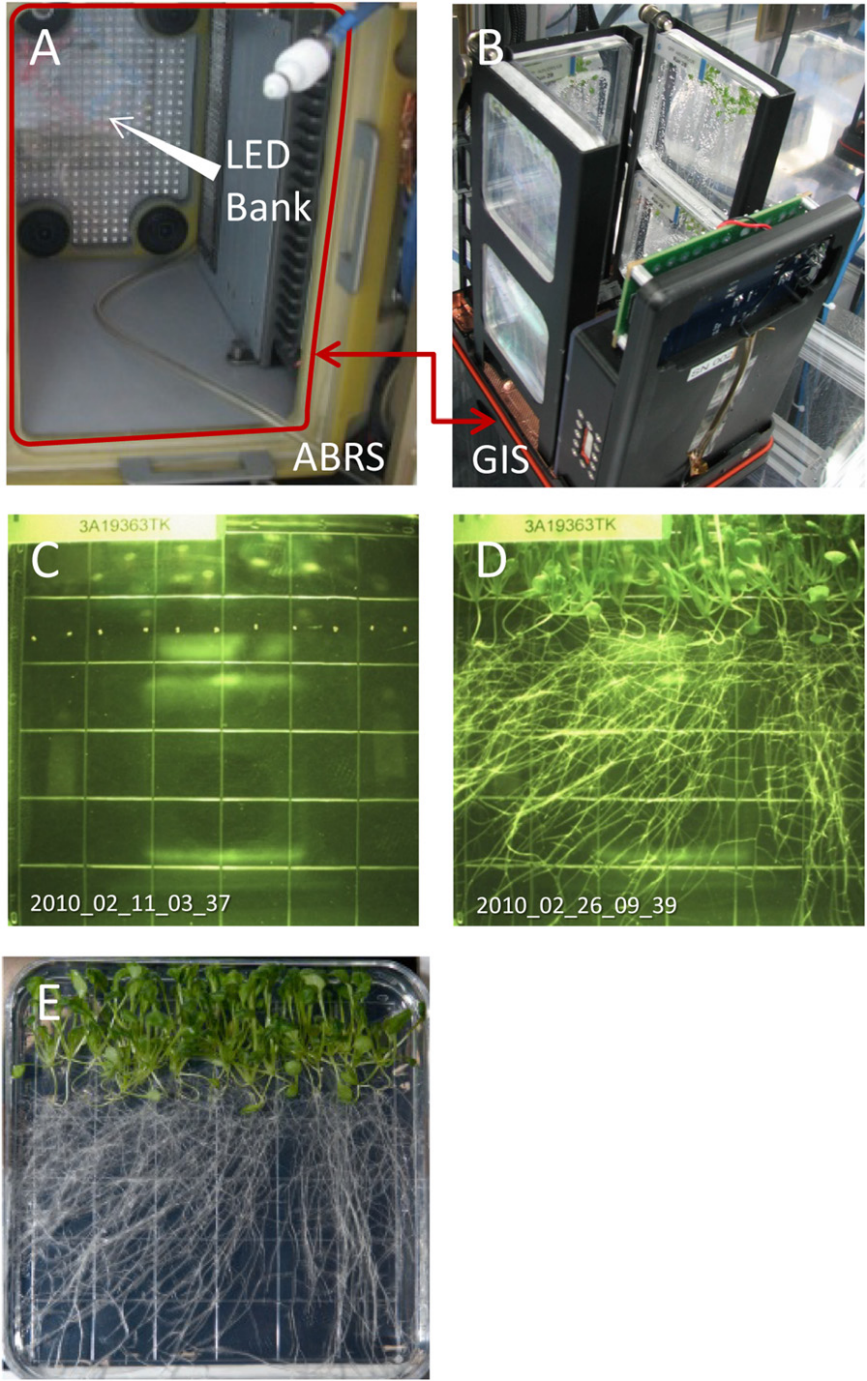
**Novel imaging hardware records distinctive patterns of growth for spaceflight and ground control plants.** The GFP Imaging System (GIS) is unique spaceflight hardware that is designed to be housed within the Advanced Biological Research System (ABRS) orbital growth chamber (**A**). The GIS contains six slots that accommodate 10 cm^2^ petri plates, three in an upper tier and three in a lower tier (**B**). The middle lower tier plate is positioned directly in front of the GIS camera and collects a set of images every 6 hours and includes an image-unique time-stamp; examples shown are from the initiation of the experiment (**C**) and at the end (**D**). Time stamps for these images are in the lower left corner. Time stamps are in the convention of year_month_day_hour_minute, thus, 1C, 2010_02_11_03_37, corresponds to 2010, February 11, 03:37 and 2010_02_26_09_39 to 2010, February 26, 09:39. The proximity to the LED light source influences the patterns of root growth between lower tier plates (**E**) and upper tier plates (**F**).

After transition to orbit and storage in a quiescent state, plates were mounted within the racks of the GFP Imaging System (GIS) hardware and then installed into the ABRS orbital growth chamber [29,30]. Germination occurred within 2 days. The LED growth lighting panel is located in the top of the ABRS chamber, which places the lighting directly above the GIS unit (Figure 1). The GIS is shown loaded with six square Petri plates, and red arrows indicate how the GIS is inserted into ABRS (Figure 1B). The imaging plate, opposite the camera in the lower tier, is designated plate #1; the remaining odd-numbered plates occupy the left and right slots of the lower tier, and the even-numbered plates populate the top tier. At 15 days the plates were removed from the GIS hardware and photographed before being harvested. The GIS imager took photographs of plate #1 every 6 hours for the duration of the experiment. One of the initial and final images taken of plate #1 is shown in Figure 1C and 1D respectively. As can be seen in 1D, the principal direction of root growth on orbit was away from the over-head light source and toward the bottom of the plate as viewed in the images. Thus the primary tropic stimulus for roots on orbit appears to be light. Further, this negatively phototropic effect on root growth was strongest in the lower tier plates, those farthest away from the light source. Although only plate #1 could be imaged continuously, selected plates from other positions were photographed at the end of the experiment as part of the astronaut harvest operations on orbit. A comparison of harvest photographs conducted at day 15 shows that a lower tier plate exhibited stronger directional growth compared to the comparable upper tier plate, which was closer to the light source and therefore received a less directional stimulus (Figure 1F). Comparable operations were executed for the ground controls within the Orbital Environmental Simulator at Kennedy Space Center. The imaging hardware facilitated the four-times-daily capture (every 6 hours) of plant growth and development on orbit and in the corresponding ground controls throughout the course of the experiment. The telemetric imaging made it possible to follow the development of the root growth patterns as they occurred, and map the changes of root orientation over time. Each image is time-stamped in the convention of year_month_day_hour_minute. Thus, the time stamp shown in Figure 1C, 2010_02_11_03_37, corresponds to 2010, February 11^th^, 03:37. The Ground Control plates were released from dormancy (by insertion into the GIS and exposing to light) precisely six days after the Flight plates were released from dormancy, thus a Flight plate image time-stamped 2010_02_19_09_4 corresponds in age (within an hour) to the Ground Control plate image 2010_02_25_10_39. This delay in the Ground control initiation allows for the programing of the Orbital Environmental Simulator with the ISS laboratory ambient environmental data collected on orbit.

**Figure 2:**
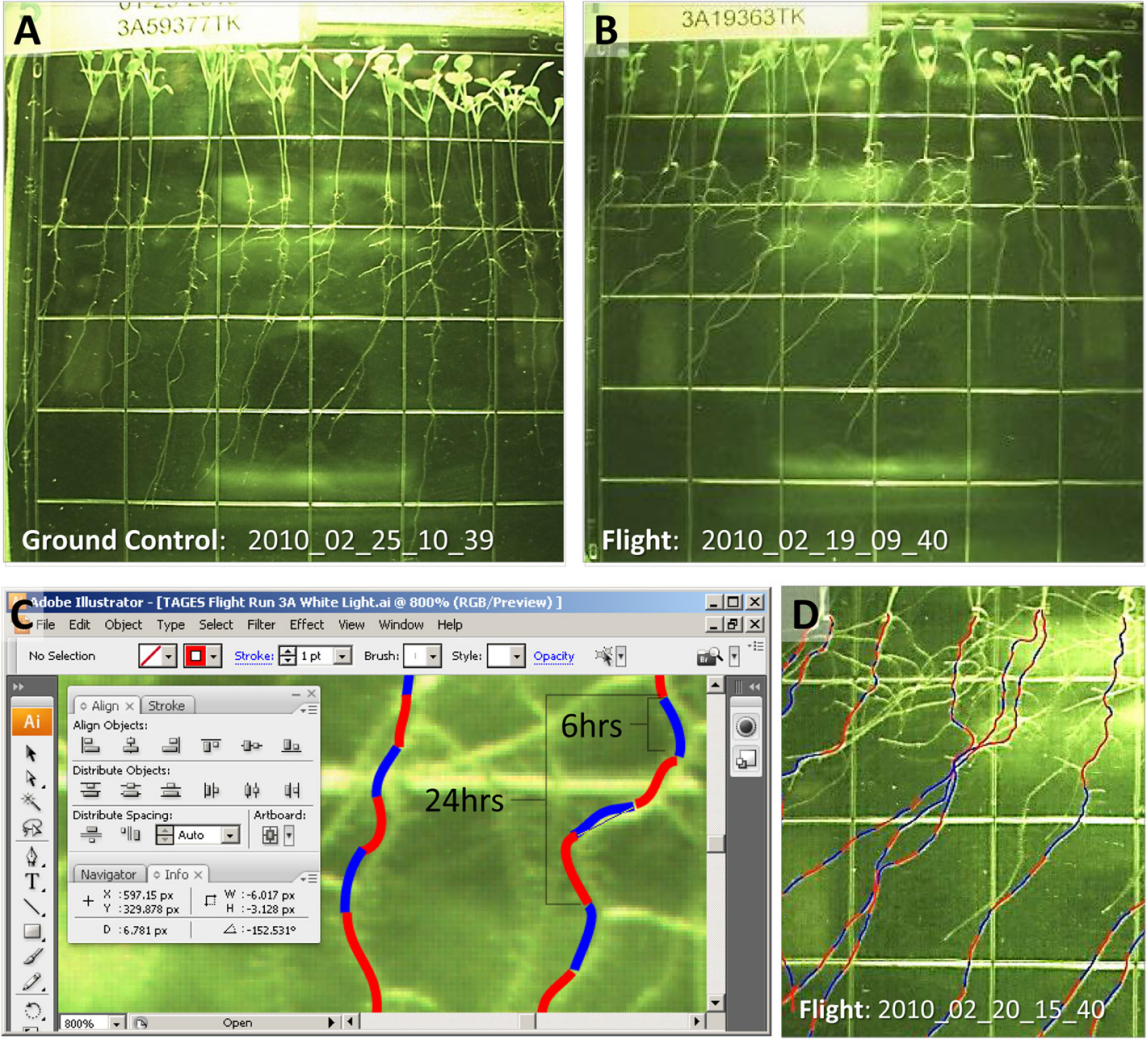
**Quantification of growth patterns with overlays provides information on the growth rates and habit.** Root growth patterns of plants 8.5 days old from the ground control (**A**) and flight experiment (**B**) were quantified with mapping options in Adobe Illustrator CS3. Numerical values were assigned to both the absolute distance grown and the degree of deviation from the vertical (**C**) which creates an overlay of data containing information on the growth rates and habit (detail in **D**).

Although plants germinated on orbit demonstrated a positive shoot phototropism and negative root phototropism that generally reflects the ground control growth patterns, the microgravity environment impacted several aspects of plant growth. These aspects were quantified with mapping options in Adobe Illustrator CS3. The analytical process is displayed in Figure 2. It can be visually seen that the growth patterns of 8.5 day old ground control (Figure 2A) and flight (Figure 2B) plants differed, but those differences can be quantified and evaluated by assigning numerical values to both the absolute distance grown and the degree of deviation from the vertical (Figure 2C). This procedure creates an overlay of data containing information on the growth rates and habit (detail in Figure 2D).

### Plants on orbit grew more slowly than comparable ground controls

**Figure 3:**
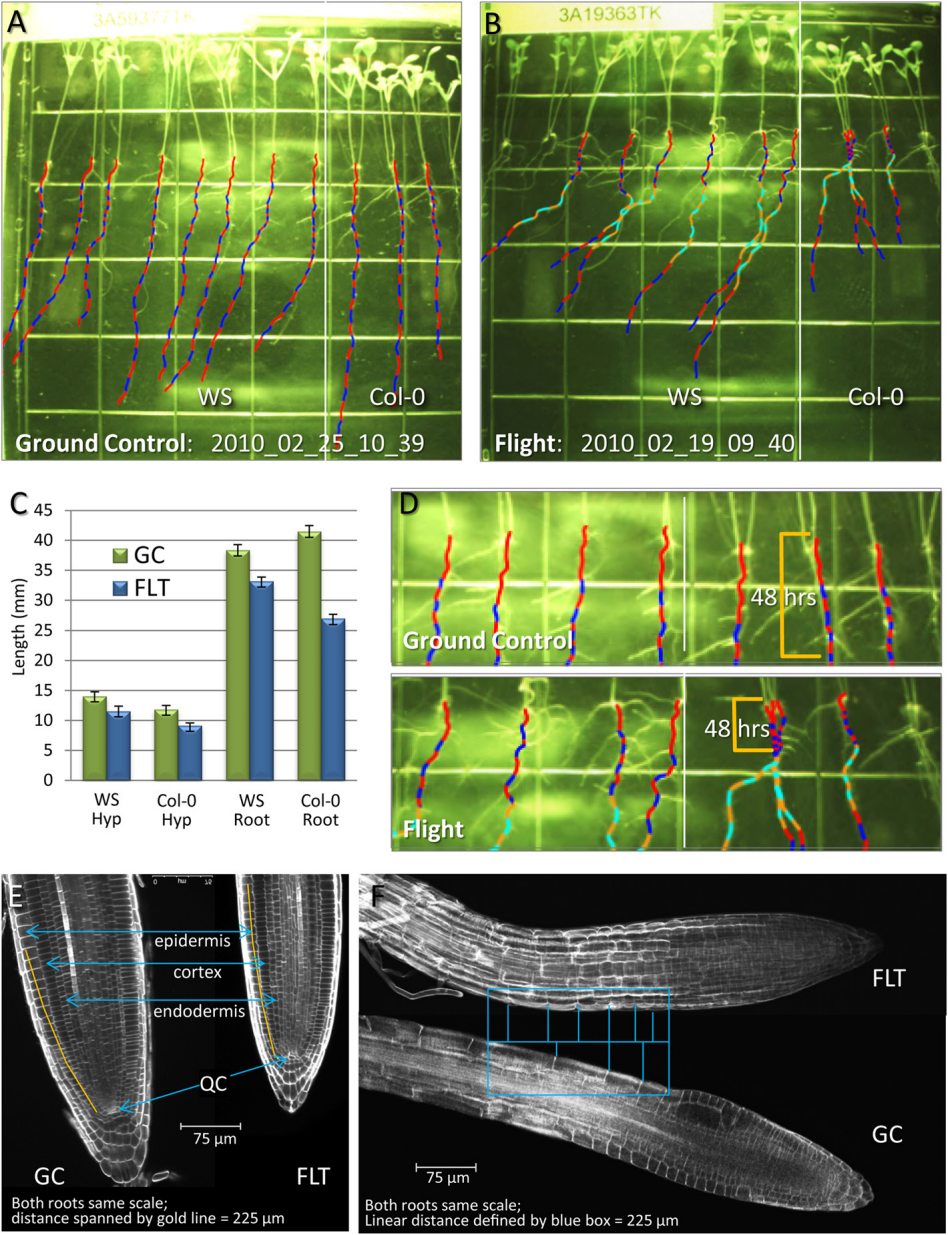
**Plants on orbit grew more slowly than comparable ground controls.** Root growth patterns of plants 8.5 days old from the ground control (**A**) and flight experiment (**B**) are shown with mapping overlays. Dotted line marks division between cultivar WS and Col-0. A colored trace was made at each 6 hour increment of growth as each plant grew, out to 8.5 days (**A, B**). Traces alternated red and blue for clarity. Sections rendered in orange and light blue represent extrapolations for missing individual photographs. Numerical values were calculated for each root and hypocotyl length (grid=13mm), and then the average values for each set of cultivars plotted with respect to treatment (**C**). Standard deviation was calculated using "n-1" method: Ground control (GC-green bars) - WS: n=11, StDev roots=0.91, StDev hypocotyls=0.70, Col-0: n=7, StDev roots=1.00, StDev hypocotyls=0.60; Flight (FLT-blue bars) - WS: n=13, StDev roots=0.67, StDev hypocotyls=0.80, Col-0: n=7, StDev roots=0.67, StDev hypocotyls=0.40. (**D**) Traced sections enlarged and the region representing the first 48hours of discernible growth highlighted with a gold bracket. (**E**) The meristematic zone in roots of ground control (GC) and flight (FLT) plants. Epidermis, cortex, endodermis and quiescent center (QC) are indicated in blue arrows. The gold line along the epidermis cell file from the quiescent center measures 225μm. (**F**) The elongation zone in roots of ground control and flight plants. The blue frame defines a region of about 225μm along the length of each root from the transition zone into the elongation zone. The cross bars in the rectangle define cell boarders in this area for each root.

Arabidopsis plants on orbit grew more slowly than comparable ground control plants. Irrespective of features inherent to spaceflight, the environmental conditions (lighting, temperature, humidity, CO_2_) experienced by the two sets of plants were identical. The ground control and flight images for 8.5 day-old plants introduced in Figure 2 were again used to generate the morphometric data presented in Figure 3. The 8.5 day images for Ground Control 2010_02_25_10_39 (Figure 3A) and Flight 2010_02_19_09_40 (Figure 3B) are shown here overlaid with the traces that define growth and direction in each 6 hour period, traces alternating red and blue for clarity (two blue, two red, per 24 hour period – see also Figure 2C). The grids on the plates measure 13mm and were used for calibration. Numerical values were calculated for each root and hypocotyl length, and then the average values for each set of cultivars plotted with respect to treatment. Ground control (GC) values are represented with green bars and Flight (FLT) values are represented with blue bars (Figure 3C). For both WS and Col-0 plants, the Flight plants showed consistently and statistically significant shorter roots and shoots. At 8.5 days, the flight plants hypocotyls were about 3 mm shorter than the ground controls in both cultivars. However, with respect to root growth, the Col-0 cultivar displayed a more dramatic lag in root development. In Col-0 the effects appear due to a slow growth phase focused in the first few days after germination, as can be seen in the very short 6-hour increments displayed in the root traces of the flight plants (Figure 3B). Sections of the traced plates are enlarged for this comparison in Figure 3D, and highlight the region representing the first 48hours of discernible growth (gold bracket). Overall, the WS cultivar averaged 5 mm shorter than the Ground Controls, while the Col-0 plants were more than 14 mm shorter after 8.5 days growth.

Confocal microscopy was used on post flight plants to image root sections of the flight and ground control plants to examine cellular morphologies that may contribute to the differential in growth rates observed between flight and ground control plants. Although the roots appear somewhat smaller, the cells of the meristematic zone in each appear to occupy the same relative space in a similar distribution (Figure 3D). Drawing a line along the epidermis cell file from the quiescent center to a distance of 225μm (yellow line overlaid on root images in 3D) it is seen that 24 cells occupied this space in the ground controls (GC) and 23 cells in the flight (FLT) root. The blue frame in Figure 3E defines a region of about 225μm along the length of each root from the transition zone into the elongation zone. The cross bars in the rectangle define cell boarders in this area for each root. In this 225μm region there were four cells in the epidermal cell file of the ground control, compared to 6 or 7 in the flight root; giving the appearance that the cells of the ground control roots were more elongated than the cells from the same region in the flight plants. This result suggests that in this growth environment, the roots of space-grown plants were shorter because the ground control roots surpassed the growth of the flight plants during the elongation phase of growth, making the differences in length a result of cell size and not cell division.

### Spaceflight roots display waving and skewing

The nature of the overhead lighting of the ABRS unit created a directional gradient of light stimuli (refer to Figure 1). This gradient set up the conditions by which directional root growth could be evaluated over time on orbit and compared to ground controls receiving the identical lighting regimen. This ability to follow root growth patterns in real time revealed the progression of developmental features, such as the patterns of growth, in addition to the final disposition of that growth. The most prominent features of Arabidopsis root growth on orbit were the adoption of a waving pattern and a dramatic skew to the right for the WS cultivar. Both cultivars recapitulated the general patterns of growth well established for their respective ecotypes in ground studies, particularly with respect to growth along a 45° inclined plane on the Earth. Growing plants along a 45° inclined plane *increases* the force of contact the root tip has with a solid surface, and in ground studies in this environment WS skews dramatically to the right, while Col-0 skews very slightly to the left. Although the orbital environment *decreases* the force of contact the root tip has with a solid surface, both cultivars grown in microgravity behave in a manner that has been attributed to a combination of gravity and the intensity of surface interaction.

**Figure 4:**
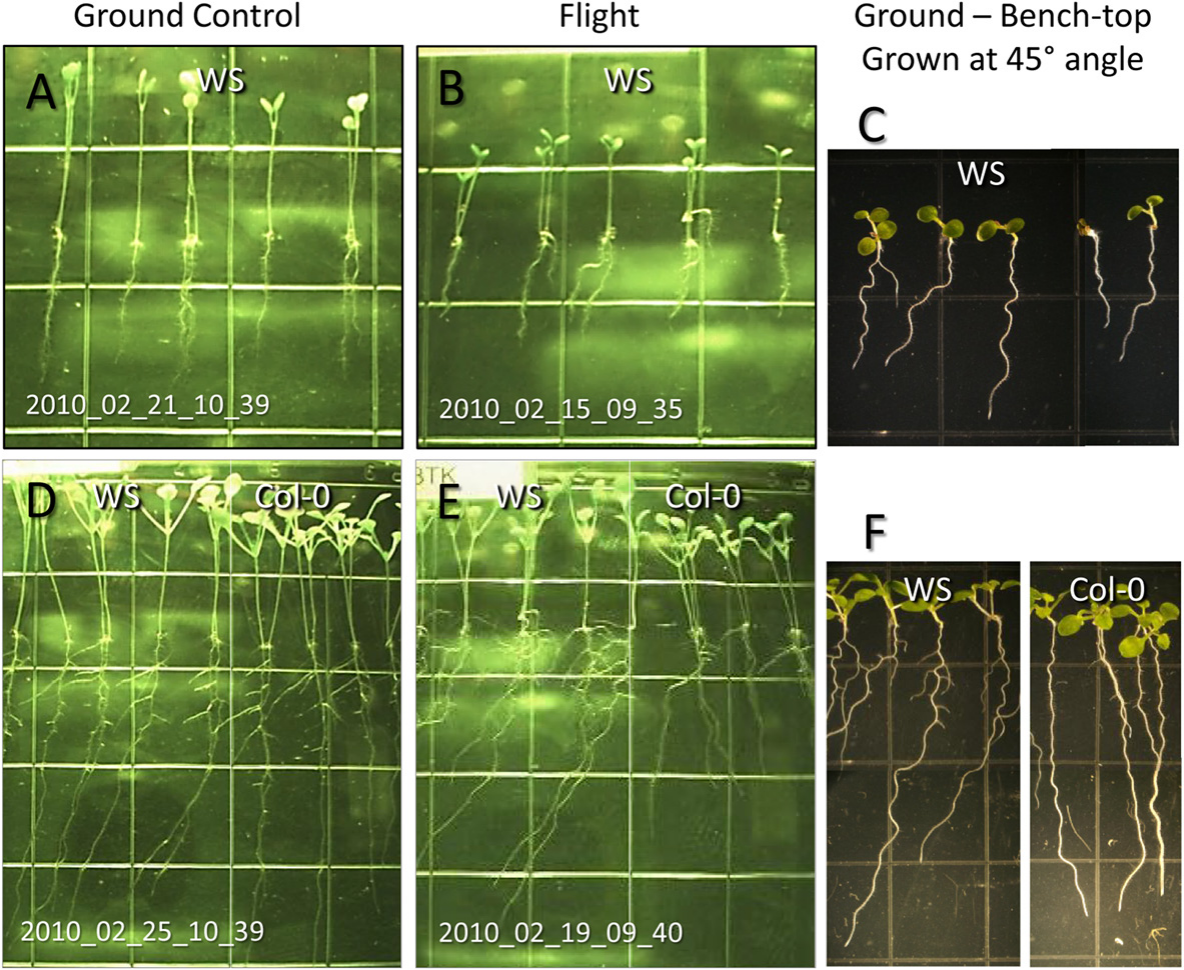
**Plants on orbit grew skew and wave, but in a novel way.** Enlargements of 5 day old WS ground control and flight plants to show near vertical root growth (**A, B**) compared to the waving behavior of 5 day old WS plants grown on 45 degree inclined agar plates in unit gravity (**C**). Enlargements of 8.5 day old WS and Col-0 ground control and flight plants to show skewing and waving patterns of root growth (**D, E**) compared to the waving behavior of 8.5 day old WS and Col-0 plants grown on 45 degree inclined agar plates in unit gravity (**F**).

Early root growth in both ground controls and flight plants of both ecotypes was almost perfectly straight away from the light, as shown in the images of 5 day ground control and flight plants in Figure 4A and 4B, respectively. However, at about day 5 the roots of the WS flight plants began to skew to the right, and by day 8 the characteristic enhanced skewing was clearly evident in the WS flight plants (Figure 4E). At first inspection, the waving of the flight plants resembled the root growth patterns characteristic of Arabidopsis gown on inclined agar plates. However, cultivar WS grown on inclined agar plates began both waving and skewing almost immediately after germination, as shown in the 5 day old WS plants in Figure 4C, and this phenomenon is well characterized in the literature [10-12,31,32]. After 8.5 days the enhanced skewing of the flight plants was clearly evident compared to the ground controls. Enlarged sections of the 8.5 day imaging plates from Figure 3 (Figure 4D and 4E) are provided for comparison next to 8.5 day old laboratory control plants grown at 45° angle to induce a similar degree of skewing as is seen in the flight plants (Figure 4F). A comparison of these three images in the bottom row illustrates that the pronounced waving typically seen with this degree of skewing in unit gravity was absent in the flight plants (Figure 4E).

**Figure 5:**
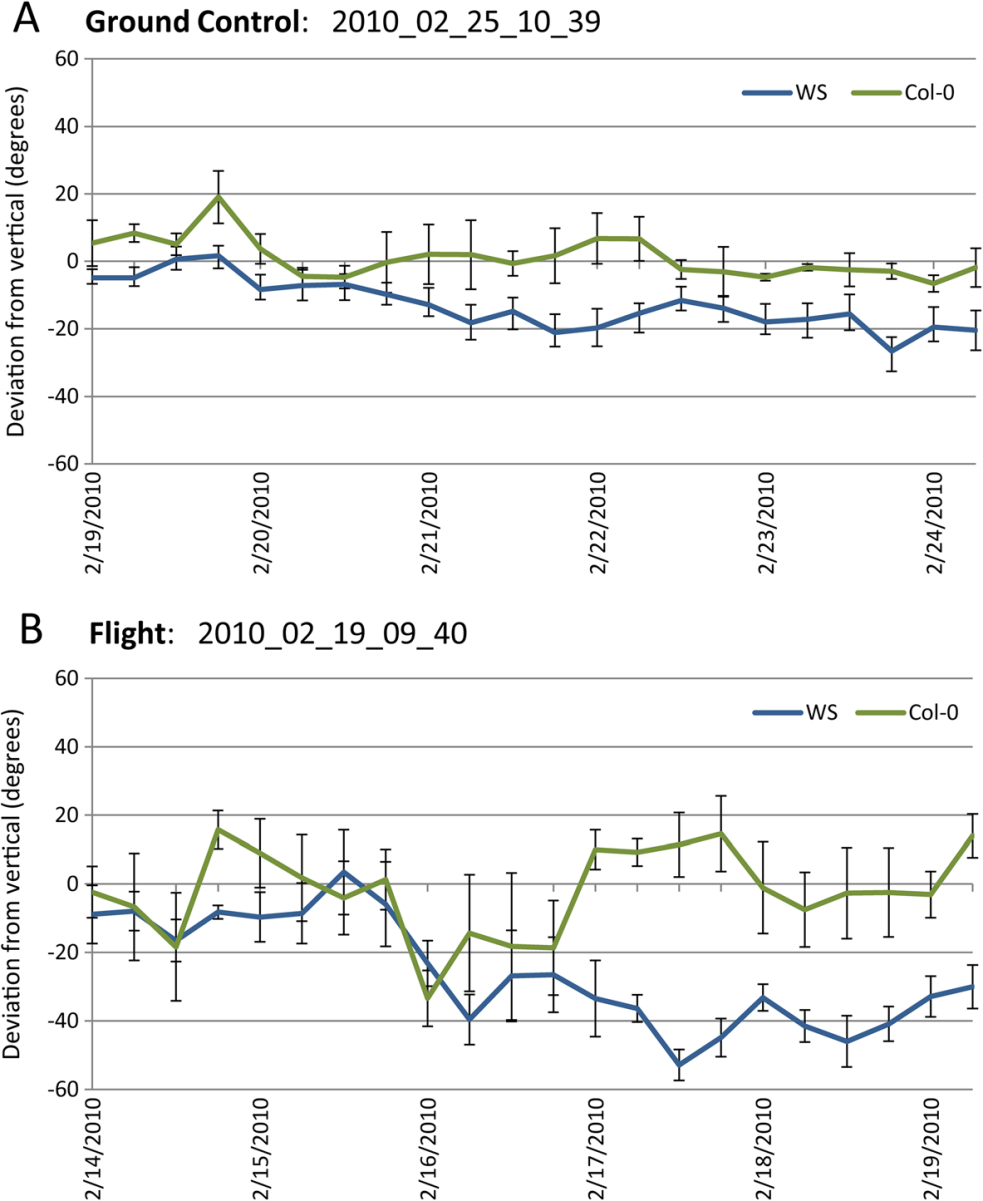
**Quantification and mapping of growth patterns.** The direction of root growth over time was quantified by taking measurements of the angle of a root segment in relation to a vertical line, with right being positive degrees and left as negative degrees. Values for each plant cultivar in ground control (**A**) and flight (**B**) in each image, spanning growth through 8.5 days, were averaged and then plotted with Microsoft Excel (see Methods for operational details). The two different cultivars represented on the imaging plate are WS (blue line) and Col-0 (green line). The y-axis shows the degree of deviation from the vertical the root presents at each 6 hour time point and the x-axis shows the corresponding dates the images were taken. Each cultivar plot line is an average taken from measurements of several roots: GC WS: 8 roots; GC Col-0: 3 roots; FLT WS: 7 roots; FLT Col-0: 4 roots. The error bars included with each time point reflect the Standard Error of the Mean.

The degree of skewing in the 8.5 day images for ground control and flight was also examined quantitatively. The traces that were used to quantify the root growth in Figure 3 were also used to generate data that show the direction and degree of deviation from the vertical of plants through about 8.5 days of growth. Three different genotypes are represented on the imaging plate: two in a WS background (seedlings on the left 2/3 view of the plate) and one in a Col-0 background (seedlings in right 1/3 view of the plate). The length and deviation data were collected for each time point and then plotted over time for clearly defined roots (Figure 5A and 5B). In both Ground Control and Flight plants, root growth of the Col-0 plants (green line) deviated only slightly from the vertical, and the two WS lines (blue line) skew to the right. What varies between ground control and flight is the degree and timing of skewing. As presented visually in Figure 4, the degree of skewing was very similar between ground control and flight until around the first five days of growth. At this inflection point, the WS ground controls transitioned to a slight skew, whereas the Flight plants took a dramatic jog to the right, transitioning from about a 10 degree skew, which they have in common with the Ground Controls, to averaging more than 40 degrees.

### Spaceflight growth patterns are not conditioned by orientation with regard to airflow, μ*g* vectors or other directional environmental factors

**Figure 6:**
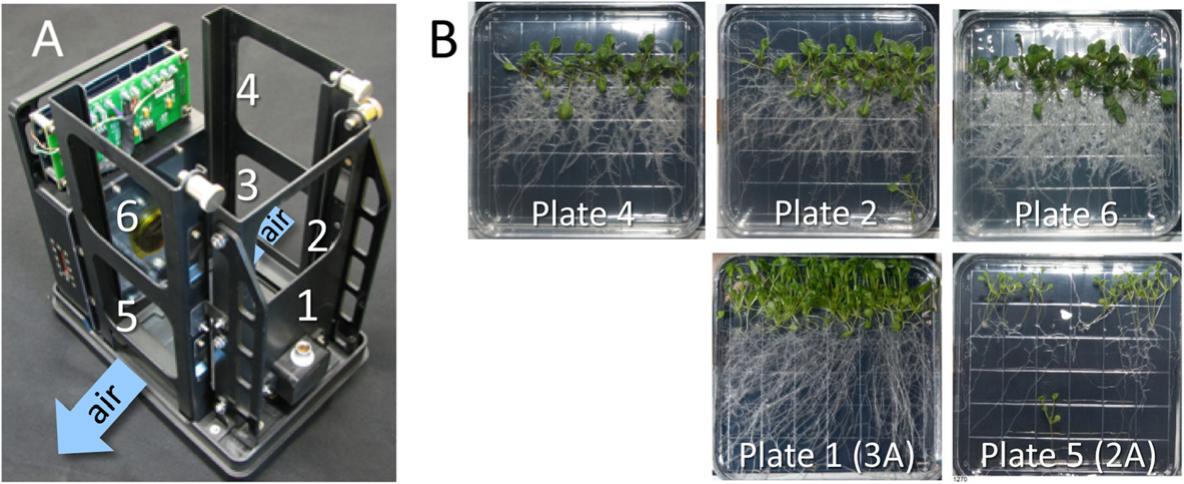
**The influence of micro-habitat on plant growth patterns.** The GIS is configured to hold six 10 cm^2^ petri plates in two tiers, numbered sequentially: bottom tier plates are numbered 1 (imaging plate opposite the camera), 3 and 5; upper tier plates are numbered 2 (directly over plate 1), 4 and 6. Air is circulated by entering the base just below plate 3 and exiting just below plate 5 (**A**). Photographs taken of the flight plants when the plates were removed from the GIS for harvest on orbit are shown in from the upper tier (Plates 4, 2 and 6) and the lower tier (plate 1 and plate 5) (**B**). Note that not all plates were harvested on orbit, and that lower tier harvest plate images are limited.

Growth patterns were the same in three different orientations within the ABRS. Orientation with respect to airflow within the ABRS and orientation with respect to ISS did not differentially affect growth patterning. The GIS fits into the ABRS unit such that the LED growth lights are directly overhead, so the upper tier plates are substantially closer to the lights than the lower tier plates (Figures 1, and 6A ). The upper tier plates received approximately 180 μmol m^2^s^-1^, while the lower tier plates received about half that. Only the bottom tier plate in position 1 of the GIS was imaged continuously, but representative plates from other positions in the ABRS/GIS were photographed prior to harvest at the maturation of the experiment. Although dense, the root growth patterns for both WS and Col-0 in the upper tier plates, where the light is more intense and less directional, appear to have been more random, whereas in the lower tier plates root growth displayed a more directed, negatively phototropic pattern. Although overall morphologies of top and bottom tier plates were different due to lighting differences, each of the upper tier plates (2, 4 and 6) and each of the lower tier plates (1 and 5 shown) displayed the same basic patterns representative of their position relative to the light source (Figure 6B). Another variable in plate position is the position around the circumference of the GIS could potentially impart a different microclimate for each plate with respect to temperature and air flow. Air is circulated by entering into the GIS from the plate-3-side of the unit and exits under the plate 5 position (Figure 6A). In addition, there is a deflector inside the GIS to redirect slightly cooler air to the back of plate 1, the imaging plate, to help keep the face of the Petri pate free of condensation. It can be easily seen in the harvest photos of the upper tier WS/Col-0 plates (plates 2, 4 and 6) that position did not appear to influence general root growth patterns (Figure 6B, top row). The same can be said of the bottom tier plates. However, since there were limited harvests of the bottom tier plates of the WS/Col-0 genotype plates per Experimental Run, the two plates shown the bottom row of Figure 6B were collected on Run 3 (plate 1; the imaging plate) and Run 2 (plate 5), of the APEX-TAGES experiment, respectively. Although plate 5 of Run 2 (launched April 5, 2010) contains the same WS/Col-0 genotype distribution as the Run 3 imaging plate, they were planted less densely and harvested at a developmental age that was 3 days younger, and thus the plants are smaller and less over-grown. Nonetheless, the *patterns* for root growth are the same – suggesting that neither the air flow nor the slight temperature differential for the imaging plate appeared to impact the patterns of root growth in the lower tier plates.

## Discussion

*Novel imaging hardware records distinctive patterns of growth for spaceflight and ground control plants*. The ability to collect growth data in real time over the life of the plant is a substantial advancement of the imaging technology available on the ISS. The ABRS/GIS hardware allowed us to follow the patterns of root and shoot growth of Arabidopsis seedlings as these structures developed over the course of the experiment. Several points along this sequence were singled out for analyses (Figures 1, 2 and 3) but a more complete continuum of these growth patterns as they develop can be seen in a two brief movies (Flight vs. Ground control) in the supplemental data. (Additional files 1 and 2).

*In the experiment described here*, *the plants on orbit grew more slowly than comparable ground controls*, *and the Col0 cultivar seemed particularly sensitive in the early stages of development*. Throughout the history of spaceflight experiments with plants, there has been no consensus on quantitative differentials in plant growth. There are examples where ground controls exhibited more extensive growth than their orbital counterparts in several species [33-35] and also examples where it was the orbital grown plants that demonstrated the more extensive growth [36-39]. It has been broadly discussed that sources for variations among plant responses, even among species, includes the diversity of flight hardware, growth systems, environmental conditions, developmental stages and cultivars used in each of these experiments [24,25]. The plants being examined here are unique in that they actually represent the only 15 day old Arabidopsis plants grown on a nutrient agar surface in the presence of a directional LED light source for an orbital experiment. Any and all of these factors – developmental stage, thigmo-stimulus, illumination – can, and do, impact the manner in which plants grow. This experiment also represents the first utilization of the ABRS hardware and GIS imaging for Arabidopsis culture. The ABRS/GIS hardware was designed to minimize the impact of confounding environmental factors on orbital habitats that have often been associated with the management of temperature and gasses in microgravity (e.g. ethylene, CO_2_, oxygen, other VOCs), and it appeared that none of these factors contributed to the patterns of plant growth in this experiment. And yet, the plants grew more slowly on orbit. A recent study demonstrated the opposite phenomenon in young dark-grown Arabidopsis in sealed containers, and showed that the basis for the elongated microgravity-grown roots was due to an elevated proliferation of cortical root meristematic cells compared to ground controls, as in a given cell file (endodermis) there was a greater number of cells per millimeter [36]. In the case of the older, light-grown Arabidopsis seedlings of this study, it was the ground control that had the longer roots. Examination of the same developmental section of the epidermis cell files of flight and ground control roots in a 225 μm region behind the quiescent center reveals similar patterns of cell proliferation in the two roots. However, the cells of the elongation zone are distinctly different in the ground control roots, being far more elongated than the cells from the same region in the flight plants. It appears then, that the plants grown in unit gravity surpassed the growth of the flight plants through enhanced elongation.

*Removing gravity from the environment facilitates the observation of intrinsic plant movement which may be masked in unit gravity*. The contribution of light as a tropic cue for root growth was broadly observed relative to the position of the six plates in the ABRS/GIS growth and imaging facility with respect to their relative distance from the light source. The patterns of plant growth vary between the upper- and lower-tier positions of the ABRS/GIS hardware, and can be directly linked to the quality of a single environmental stimulus: light. The management of growth light illumination was balanced to provide adequate illumination for the lower tier imaging plants, while not overwhelming the plants in the upper tier. This middle ground of illumination intensity resulted in an interesting division of growth patterning between the two latitudes. In the lower- tier plates the gradient of light intensity established by the over-head illumination in the ABRS unit had the effect to enhance the tropic effect of light on these plates. The hypocotyls elongated towards the light, while the roots grew primarily away from the light, operationally “down” the surface of the plate. Although the intensity of illumination at this position (80 μmol m^-2^s^-1^) is slightly less than ideal, this lighting environment facilitated a clear resolution of the other factors that influence the pattern of root growth, particularity when freed from the overwhelming positive tropism imposed by unit gravity. The upper- tier plates are situated very close to the overhead lights and received about 180 μmol m^-2^s^-1^. The consequence of this close proximity was that there was very little in the way of a “vertical” gradient of light stimulus established in the upper- tier plates, and the underlying inherent growth patterns are more difficult to resolve on the background of less tropically-directed growth of roots and shoots (Figure 1).

*The patterns of waving and skewing seen on orbit clearly demonstrate that gravity is not an essential component of the mechanism that drives these configurations of root growth*. It has been well established that the roots of certain cultivars of Arabidopsis naturally skew to the right when growing along a resistive surface. In addition, when such a growing surface is inclined at an angle of 30 – 45 degrees, roots grow in waving patterns as they navigate the surface [6,8-12,31,32,40]. The underlying reasons for these morphological characteristic of some Arabidopsis cultivars is not completely understood (Reviewed in: [6,8]) but there is every indication that it is at least linked to the manner in which the root samples its environment (e.g. [41,42]). It has been suggested that that waving and skewing are primarily a result of interactions between either gravitropism and the tactile interaction with a surface (thigmotropism) [9,11,43] or gravitropism and circumnutation [9,40], as well as being influenced by circadian rhythm, ionic and nutrient environment and other biotic/abiotic influences [44-47]. And although the specific metabolic drivers are not fully known, comparisons of differential expression among cultivars has suggested a number of here-to-fore unknown candidate genes that may contribute to cultivar-specific phenotypes, as well as confirming several known to be involved with skewing and waving [48]. Regardless of the underlying influences, the consensus that gravity contributes to waving and skewing deserves reconsideration.

*Although gravity is not required for skewing or waving*, *light contributes to the characteristic of waving and skewing on orbit*. It has long been known that although gravity amplifies the subtle oscillations of plant movement, such as circumnutation, plants can exercise these movements in the absence of gravity. Indeed, many of the early orbital plant experiments focused on this demonstration (e.g. [19,49,50]). However, it has also been shown that gravity sensing cells and systems are important for the execution of these movements [51,52]. On earth, the contribution of light/phototropism is minimal compared to the impact of gravity on directional root growth. In the spaceflight environment of the present study the upper tier plates received an intense distribution of light from a relatively large light area. In these plates, root growth was compact and confined to the middle third of the possible growing area of the plate (Figures 1 and 6). However, the lower tier plates received a more directional and attenuated light intensity. Root growth on these plates were more negatively phototropic and visually mimicked most features of the positive gravitropic growth of comparable ground controls (e.g. Figures 1 and 6)

Although the flight plant roots looked very similar to plants grown on inclined agar plates, there are some growth features that were missing in the spaceflight plants. Spaceflight roots were slow to initiate significant skewing. In this way, they most closely mimicked the natural tendency of WS to skew a bit to the right when grown vertically in unit gravity, as opposed to on inclined plates, and this characteristic can be seen visually and in the quantification of the patterns shown in Figure 5. At about day 5, when the roots were 7–10 mm long, the roots began a more substantial jog to their right. This new angle of growth does closely mimic what is seen for WS grown on agar plates tilted to 45°, with one exception: the wavy phenotype that is a hallmark of inclined plate growth assays is subtle when compared to the degree of waving that is associated with plants exhibiting the degree of skewing shown by the flight plants. These data reinforce the paradigm that skewing and waving represent two separate phenomena and can be disconnected, and the further disconnection of these features of root growth in the flight plants indicates that gravity is not strictly required for either.

There has been much discussion in the literature as to why Arabidopsis roots grow in a wavy pattern on inclined agar plates, and the discussion can be summarized with a quote from an early review: “*This waving behaviour has been interpreted as representing a gravitropismdependent thigmotropic response*. *We argue instead that this root waving represents primarily a flattened spiral growth pattern resulting from circumnutation and gravitropism*” Simmons et al. 1995a [40]. Recent work demonstrates that there are numerous mutations that affect phenotypes of waving but not skewing, and vice versa [8 and supplimetal data therein] and a number of these mutants have additional phenotypes that also match the orbital phenotype we observed in this study, like reduced root elongation [32,53]. More recently a combination of quantitative trait loci (QTL) explorations and microarray analysis of the differential expression among natural cultivars with distinct skewing phenotypes has identified a region on chromosome 2 which contributes to skewing phenotypes [48]. In all cases, the consensus is that gravitropism is the directional driver in the skewing and waving phenotype, and it does play the major role on earth. However, gravity is not required. Right-handed skewing in root growth appears to be an inherent feature of many Arabidopsis ecotypes, and can be seen even in culture conditions lacking both light and gravity [16]. Further, the data presented here illustrate that classic waving and skewing patterns, characteristic of presumed gravitropism-driven interactions with a solid surface, happen in the absence of gravity. This strongly suggests that it is not the force of gravity-driven interactions with surfaces that drive skewing, and that the role of gravity is more in directional cuing, which on orbit can be supplied by the light source.

## Conclusions

Although plants use gravity as an orienting tropism on Earth’s surface, it is clear that gravity is neither essential for root orientation, nor the only factor influencing the patterns of root growth. In the absence of gravity but the presence of light, roots remain strongly negatively phototropic. Further, roots wave and skew in directions characteristic of their genotypes. Differences in cultivars are more pronounced when on orbit in the absence of gravity. Although both WS and Col-0 cultivars are clearly negatively phototropic in microgravity they behave differently: WS skews strongly to the right in the absence of gravity, while cultivar Col-0 exhibits minor skewing to the left. These results speak directly to the role of gravity in the directional movement of roots.

The primary idea for the evolutionary role of waving and skewing is that it confers an adaptive advantage in obstacle avoidance during growth. This idea intersects with circumnutation and thigmotropism, and of course, there is room for considerable overlap in the phenomenology. The genes that govern all of these responses are still being revealed, and we hypothesize from other work with plant responses in novel spaceflight environments that when an organism is sampling its surroundings in the absence of normal feedback, it may respond in a seemingly inappropriate manner [22,28,54]. Does a root need to wave and skew to avoid an obstacle in microgravity? No. But in the absence of the overwhelming driver of gravity experienced on earth, it seems that other features of the environment set up the responses to ensure that a root *does* at least grow away from the seed, and thereby attempt to enhance its chances of finding sufficient water and nutrients to ensure its survival.

## Methods

### Plant Material

The three lines of *Arabidopsis thaliana* used in this experiment were: UB::GFP (35sCaMV constitutive promoter, ecotype WS [55]), Adh::GFP (alcohol dehydrogenase promoter, ecotype WS [56]) and DR5::GFP (synthetic auxin response element; gift of T. Guilfoyle, ecotype Col-0 [57]). Seeds, and the seeded plates, were prepared in such a way as to maintain dormancy until the initiation of the experiment on orbit. Seeds were surface sterilized with 75% ethanol for 10 minutes, dried on sterile filter paper and stored at 4°C until use. Immediately before planting, a small amount of seed was suspended in sterile water and then the seeds were dispersed individually to the surface of a 100mm^2^ solid media plates [56]. The plate was then immediately wrapped in light-tight black cloth (Duvatyne) and stored at room temperature until launch. After launch (Shuttle launch STS-130 - 09:14, 8 February 2010) and transition to the International Space Station (ISS), the wrapped plates were stowed at ambient temperature for a little less than three days until the initiation of the experiment on orbit. The seeds remained dormant until activated by exposure to light on February 11^th^, 2010.

### Plant growth in imaging hardware

Both the flight and ground control components of the experiment were grown in specialized growth chamber units developed by Kennedy Space Center referred to as the ABRS (Advanced Biological Research System) growth chamber [30,58,59]. Seeded plates were unwrapped from their light-tight coverings and installed in the ABRS/GIS plant growth chamber and imaging hardware. The GIS is designed to hold six 10 cm square plates: one in the direct line of the imaging camera, plus five additional plates (Figure 1) [29]. The Imaging camera is designed to collect both white light and fluorescent images, and as such, incorporates a GFP imaging filter is an integral component of the camera. Thus, even the “white light” (image taken in the absence of the GFP excitation illumination and in the presence of the grow-light immunization) contains a greenish cast, and it is difficult discern colors (such as the true green of the leaf material). Nonetheless, the white light images provide a clear record of root growth and morphology over the duration of the experiment. The Ground Control runs were conducted within a complete, second ABRS unit that was housed in the Orbital Environmental Simulator (OES) chamber in the Spaceflight Life Sciences Laboratory at Kennedy Space Center. The Ground Control was initiated with a precise 6 day delay to enable the OES environment to be programed with those environmental conditions that can be replicated on the ground, such as cabin temperature and CO_2_ levels, taken from ISS telemetry.

### Configurations of the ABRS and GIS

ABRS is composed of two independently programmable Environmental Research Chambers (ERCs), one of which housed the Experiment Unique Equipment referred to as the Green Fluorescent Protein Imaging System. Growth lighting was provided by Light Emitting Diode (LED) arrays composed of 303 LEDs arranged in banks to facilitate Pulse Width Modulation control of light intensity from 50 to 300 μmol m^-2^s^-1^, and provided a spectrum of photosynthetically available light (peaks at 470 and 660nm) plus minor green and white light components. The ABRS environment was controlled for temperature and CO^2^ concentration. The environmental set points for the experiment presented here were: lighting at 180 μmol m^-2^s^-1^ (measured at the top of the upper tier plates, it was about half this at the level of the imaging plate, 12cm below this mark), temperature at 23°C and CO_2_ concentration at 3000ppm.

The GIS holds six standard 100 mm square Petri plates (Fisher Scientific) in two tiers. Plate in position 1 of the bottom tier plates is housed directly across from an imaging camera (Figure 1). The GIS can provide 470nm LED illumination, and the camera contains a long pass filter (505nm) for collecting GFP-expression images. The camera can also collect images during illumination from the growth lights, creating “white light” images, such as were used in the growth analyses presented here. Images were taken every six hours and stored to an SD card housed in the unit. The images were also downlinked daily to the ground.

The ABRS/GIS Flight unit was housed in the Express Rack on the ISS, and the Ground Control unit was housed in the Orbital Environmental Simulator (OES) at Kennedy Space Center. The OES was programed with the same environmental conditions experienced on orbit, and the Ground Control was executed with a 6 day delay. The configurations of the Flight and Ground Control ABRS/GIS units were identical, although the aim of the imaging camera in the Ground Control GIS results in the capture of the plate image that is shifted about 3 mm compared to the Flight unit. This imaging difference does not compromise data collection, but may be noticed in several of the figures where Ground Control and Flight images are aligned (e.g. Figures 1 and 4).

### Confocal microscopy

Samples from the Flight and Ground Control plants that were preserved in RNAlater were washed with distilled water and then stained with calcofluor white to label the cell walls [60,61]. Stained tissues were imaged on a Zeiss Pascal LSM5 Confocal Laser Scanning Microscope.

### Mapping and quantification of root growth patterns

Images from the GIS imager were taken in intervals of six hours. Those images were used in succession to trace the segment of root growth over that period of time for each interval using Adobe Illustrator CS3. The original 16-bit TIFF GIS files were first converted into 8-bit JPEGs using David’s Batch Process extension for GNU Image Manipulation Program, using 2x2 sampling and no loss in quality. The JPEG images were then imported into Adobe Illustrator CS3 as separate layers. The layers in Illustrator were labeled with the original timestamp found in the filename of the image. The segment traces were obtained by selecting a starting point image with some root growth already present, making it the only visible layer out of all of the imported image layers, and using a Wacom Intuos2 tablet to freehand draw a vector line over the existing root segment. Manual tracing was chosen as the best viable option, since there was difficulty using OCR (Optical Character Recognition) software to find the roots due to the monochrome nature of the images, the gridlines, and glare from the imager itself. The manual vector trace layer was kept at the foreground at all times. At the completion of the first trace layer, the next image in the layer sequence was made visible, and the manual trace resumed on the top vector trace layer. This procedure was repeated for all images in the set. The end result was an overlay of the cumulative manual traces over the final image, which provided a snapshot of all of the growth periods given by each image at the same time.

The direction of root growth over time was quantified by taking measurements of the angle of a root segment in relation to a vertical line, with right being positive degrees and left as negative degrees. This operation was completed using Illustrator’s native “Measure Tool” and then entering the angle (degrees) and length (pixels) captured from the Illustrator Information window into a table in Microsoft Excel. Individual measurements were recorded for every root segment drawn (Figure 2). In instances where the an image in the sequence was unavailable, the total root length from one image to the next was divided by the number of missing intervals in order to obtain an estimate of the growth over those time periods. The angles of root growth were then measured from those estimated segments. Estimated segments are indicated by a distinct color in the composite image. All data were graphed using Microsoft Excel. The final graphic representation of root skewing was obtained by taking the average length and angle of the comparable segment of all individual roots of the same genotype (Figure 5).

### Skewing and waving assays in unit gravity

The WS and Col-0 lines used in the flight experiment were also grown on a 45 degree incline to evaluate the waving and skewing behavior of these lines in the laboratory. Plates were prepared as described above for the spaceflight experiments, using the same composition of phytagel media (not the harder, 1.5% agar media typically used in waving assays). Half of the plates were allowed to grow vertically and the remainder inclined to present a 45 degree surface for the emerging root. The plates were grown in this configuration for 10 days under standard laboratory growth conditions. The plates were photographed through the front of the plate on day 5 and midway through day 8.

## Competing interests

The authors have no competing interests related to this manuscript.

## Authors’ contributions

ALP and RJF contributed equally to the design, conduct and analysis of the experiments. CEA handled imagining data for the mapping and quantification of root growth patterns to create traces in Figure 5 and movies for supplemental data. All authors read and approved the final manuscript.

## Supplementary Material

Additional file 1 **Plate 1, Run 3A – growth over time in ABRS/GIS in Flight.** The collection of images in the Flight plate used to generate the traces and skewing data of Figures 3 and 5 are presented as a movie to demonstrate to progression of plant growth in microgravity. Click here for file

Additional file 2 **Plate 1, Run 3A – growth over time in ABRS/GIS in Ground Control.** The collection of images in the Ground Control plate used to generate the traces and skewing data of Figures 3 and 5 are presented as a movie to demonstrate to progression of plant growth in microgravity. Click here for file
